# Transcranial Doppler microemboli and acute brain injury in extracorporeal membrane oxygenation: A prospective observational study

**DOI:** 10.1016/j.xjtc.2022.07.026

**Published:** 2022-08-20

**Authors:** Giorgio Caturegli, Shrey Kapoor, Vladimir Ponomarev, Bo Soo Kim, Glenn J.R. Whitman, Wendy Ziai, Sung-Min Cho, HERALD (Hopkins Exploration, Research, and Advancement in Life Support Devices) Investigators, Lucy Q. Zhang, Yunis Mayasi, Aaron Gusdon, Bahattin Ergin, Steven Keller, Matthew Acton, Hannah Rando, Diane Alejo, Kate Calligy, Scott Anderson, Benjamin Shou, Pedro A. Mendez-Tellez, Henry Chang, Marc Sussman, Christopher Wilcox, Patricia Brown, Anna Peeler

**Affiliations:** a Division of Neurosciences Critical Care, Departments of Neurology, Neurosurgery, Anesthesiology and Critical Care Medicine, Johns Hopkins University School of Medicine, Baltimore, Md; b Division of Pulmonary and Critical Care Medicine, Department of Surgery, Johns Hopkins University School of Medicine, Baltimore, Md; c Division of Cardiac Surgery, Department of Surgery, Johns Hopkins University School of Medicine, Baltimore, Md

**Keywords:** ECMO, extracorporeal membrane oxygenation, TCD, transcranial Doppler, brain injury, stroke, MES, emboli, ABI, acute brain injury, ACA, anterior cerebral artery, aPTT, activated partial thromboplastin time, BA, basilar artery, ECMO, extracorporeal membrane oxygenation, HIBI, hypoxic ischemic brain injury, ICA, internal carotid artery, MCA, middle cerebral artery, MES, microembolic signal, TCD, transcranial Doppler, VA, venoarterial, VV, venovenous, VrA, vertebral artery

## Abstract

**Objective:**

Extracorporeal membrane oxygenation (ECMO) carries a high morbidity of acute brain injury (ABI) with resultant mortality increase. Transcranial Doppler (TCD) allows real-time characterization of regional cerebral hemodynamics, but limited data exist on the interpretation of microembolic signals (MES) in ECMO.

**Methods:**

This prospective cohort study was conducted at a single tertiary care center, November 2017 through February 2022, and included all adult patients receiving venoarterial (VA) and venovenous (VV) ECMO undergoing TCD examinations, which all included MES monitoring.

**Results:**

Of 145 patients on ECMO who underwent at least 1 TCD examination, 100 (68.9%) patients on VA-ECMO received 187 examinations whereas 45 (31.1%) patients on VV-ECMO received 65 examinations (*P* = .81). MES were observed in 35 (35.0%) patients on VA-ECMO and 2 (4.7%) patients on VV-ECMO (*P* < .001), corresponding to 46 (24.6%) and 2 (3.1%) TCD examinations, respectively. MES were present in 29.4% of patients on VA-ECMO without additional cardiac support, compared with 38.1% with intra-aortic balloon pump and 57.1% with left ventricular assist device, but these differences were not statistically significant (*P* = .39; *P* = .20, respectively). Presence or number of MES was not associated with VA-ECMO cannulation mode (23.4% MES presence in peripheral cannulation vs 25.8% in central cannulation, *P* = .80). In both VA- and VV-ECMO, MES presence or number was not associated with presence of clot or fibrin in the ECMO circuit or with any studied hemodynamic, laboratory, or ECMO parameters at the time of TCD. ABI occurred in 38% and 31.1% of patients on VA- and VV-ECMO, respectively. In multivariable logistic regression analyses, neither ABI nor a composite outcome of arterial thromboembolic events was associated with presence or number of MES in VA- ECMO.

**Conclusions:**

TCD analysis in a large cohort of patients on ECMO demonstrates a significant number of MES, especially in patients on VA-ECMO with intra-aortic balloon pump, and/or left ventricular assist device. However, clinical associations and significance of TCD MES remain unresolved and warrant further correlation with systematic imaging and long-term neurologic follow-up.

**Figure:**
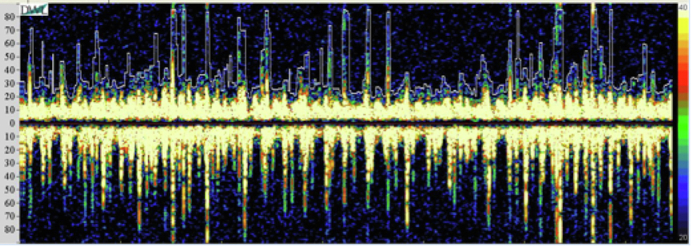
High microembolic signals in the left middle cerebral artery in VA-ECMO patient.

**Central Message**

Transcranial Doppler ultrasound in a large cohort of extracorporeal membrane oxygenation patients shows a high number of microembolic signals, but their clinical significance remains unresolved.

**Perspective**

Extracorporeal membrane oxygenation carries a high risk of acute brain injury with a resultant increase in mortality. Transcranial Doppler can detect microembolic signals in real-time, but limited data exist on their interpretation in ECMO. Here we report preliminary results of our prospective observational cohort study on MES in ECMO and their associations with neurologic outcomes.

Extracorporeal membrane oxygenation (ECMO) is a form of temporary mechanical circulatory support indicated for medically refractory pulmonary and/or cardiac failure.^1^ The presence of acute brain injury (ABI), such as ischemic stroke, intracerebral hemorrhage, and hypoxic ischemic brain injury (HIBI), approximately doubles an already-high mortality risk in patients receiving ECMO.^2^^,^^3^ ABI rates of 5% to 10% from registry studies are likely underestimated, due to lack of standardized neuromonitoring and the challenges of neuroimaging patients on ECMO.^2^, ^3^, ^4^, ^5^ This underestimation is supported by the much greater prevalence (up to 85%) described in pathologic^6^, ^7^, ^8^ and prospective clinical studies.^9^, ^10^, ^11^ Embolism propagated from a cardiac or circuit source is a further potential source of ABI.

Transcranial Doppler (TCD) is a noninvasive, low-risk, bedside technique that identifies cerebral microembolic signals (MES) in real-time. Due to technical requirements of TCD and limited experience in patients receiving ECMO, data on MES and their clinical significance in this population remain understudied. At present, one study has examined MES in detail longitudinally with 248 TCD examinations in an ECMO cohort of 53 patients,^12^ describing the presence of MES in 81.8% and 26.2% in patients receiving VA- and VV-ECMO, respectively. Among many studied clinical parameters and outcomes, the only significant association was an inverse correlation between cardiac ejection fraction and the number of MES in VA-ECMO. In the present study, we report results of our prospective observational cohort study of TCD MES and its association with neurological outcome. We hypothesized that a more frequent presence and/or greater number of MES would be associated with a greater frequency of ABI and arterial thrombotic events in ECMO.

## Methods

### Data Availability

Data are available upon reasonable request and completion of a data use agreement at https://www.hopkinsmedicine.org/heart_vascular_institute/cardiovascular-research/by_laboratory/cardiac_surgery_research_lab/our_team.html.

### Study Design

All patients receiving ECMO who were cannulated at a single tertiary care medical center are included in a prospective cohort that undergoes a neurologic monitoring protocol, including TCD and a Neurocritical Care consultation.^11^ Electronic health records were reviewed for all adult patients supported on ECMO undergoing TCD examinations in the study period from November 2017 through February 2022.

### Study Participants

All adult (>18 year old) patients who received ECMO were included. Only a patient's first ECMO run was included, to minimize potential bias resulting from severe illness. Also excluded were patients with absent temporal windows or those who did not receive TCD, for instance, due to early death or noncompliance with the standardized neuromonitoring protocol, typically due to logistical challenges in the early phase of the coronavirus disease 2019 pandemic. This study was approved by the Johns Hopkins University Institutional Review Board (IRB00216321, approved October 22, 2019), and need for consent was waived as an observational study.

### Data Collection and Definitions

Pre-ECMO characteristics including demographics, medical history, and precannulation neurologic function (Glasgow Coma Scale) were collected. ECMO variables included indication, cannulation method (central [aortic] vs peripheral eg, femoral–femoral, femoral–internal jugular, internal jugular dual lumen), ECMO flow (L/min), and ECMO pump speed (rpm). A dedicated perfusion team assessed the ECMO circuit multiple times daily to identify the presence, position (inlet vs outlet), and distance (greater or less than 2 inches from pump head) of fibrin and clot. Selected hemodynamic parameters (systolic blood pressure, diastolic blood pressure, mean arterial pressure) and laboratory findings (hemoglobin/hematocrit, platelet count, fibrinogen, arterial blood gas, activated partial thromboplastin time [aPTT], international normalized ratio) were collected at the closest-available time to each TCD. These parameters were selected based on physiological relevance to microembolic signals.^13^^,^^14^

### TCD Protocol and Variables

Serial TCD (DWL; Compumedics DWL) examinations were performed for patients on ECMO. As permitted by duration of ECMO support, 3 TCDs were scheduled routinely for all patients on ECMO at ECMO day 1, and between days 3-5 and days 7-10.^11^ TCDs were performed by 1 of 2 experienced, licensed TCD technologists and reviewed by a neurocritical care physician with expertise in TCD interpretation. Additional examinations were performed as clinically indicated, such as for positive MES, subarachnoid hemorrhage monitoring, or brain death evaluation. All TCD examinations were performed by 1 of 2 registered vascular technologists, using standard TCD protocols^15^ to manually assess the number of MES manually for 30 minutes in every TCD examination. Anterior (internal carotid artery [ICA–C1 segment], middle cerebral [MCA–M1 segment], anterior cerebral artery [ACA–A1 segment]) and posterior (vertebral artery [VrA], basilar artery [BA]) intracranial vessels were assessed.

### Definitions and Outcomes

The primary outcome was ABI, composed of ischemic stroke, HIBI, intracerebral hemorrhage, subdural hemorrhage, subarachnoid hemorrhage, or brain death. Additional analysis targeted ABI subtype, particularly ischemic stroke. Secondary outcomes were systemic thromboembolic events, both arterial (ischemic stroke, HIBI, pulmonary embolism, intracardiac thrombus, arterial cannula clot) or venous (deep venous thrombosis, heparin-induced thrombocytopenia, disseminated intravascular coagulation).

When correlating TCD-detected MES with clinical outcomes, positive MES was defined as the presence of MES in any vessel on any examination for a given patient while on ECMO. The number of MES on each examination was graded as previously described: negative (no MES), mild (1-20 MES), moderate (21-99 MES), and severe (≥100 MES and the “curtain” effect).^12^ Neurologic outcomes including modified Rankin scale score at discharge were assessed by a neurologist-led neurocritical care team as part of a neuromonitoring protocol.

### Statistical Analysis

Primary outcomes were described as proportions for categorical variables and as median and interquartile range for continuous variables. Associations between MES presence and grade with ABI and other physiologic parameters were tested using the χ^2^ test, Fisher exact test, or the Mann–Whitney *U* test as appropriate. Multivariable logistic regression, including variables with *P* values less than .20 on univariate analysis, was performed to test the association of presence of MES with the outcome composite arterial thromboembolic events (ischemic stroke, HIBI, pulmonary embolism, intracardiac thrombus, arterial cannula clot). Analyses were conducted in STATA 16.0 (StataCorp LLC).

## Results

### Study Participant Characteristics

**Figure 1:**
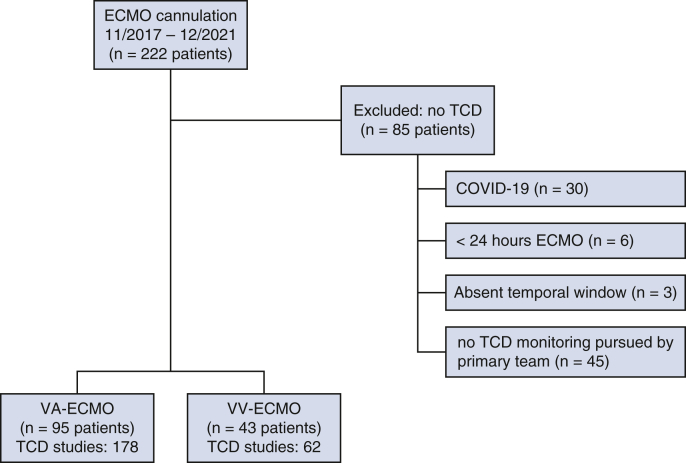
Study design and participant selection. Patients receiving at least 1 transcranial Doppler (*TCD*) examination were selected from a prospective cohort of venoarterial (*VA*) and venovenous (*VV*) extracorporeal membrane oxygenation (*ECMO*) at a single tertiary care center.

**Figure 2:**
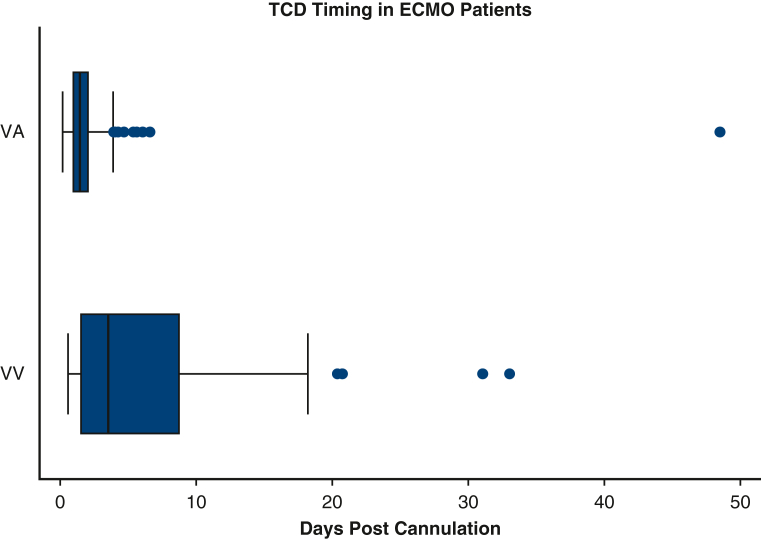
Number and timing of transcranial Doppler (*TCD*) examinations with respect to cannulation among included patients on venoarterial (*VA*)- and venovenous (*VV*)-extracorporeal membrane oxygenation (*ECMO*).

During the study period, 229 consecutive patients received ECMO support, of whom 145 (63.3%) underwent at least 1 TCD examination: 100 (68.9%) patients receiving VA-ECMO underwent a total of 187 examinations, whereas 45 (31.1%) patients receiving VV-ECMO underwent a total of 65 examinations (Figure 1). Reasons for not completing a TCD examination are shown in Figure 1. Median time from ECMO cannulation to first TCD examination was 1.4 (0.9-2.1) days for VA- and 3.5 (1.5-8.8) days for VV-ECMO (*P* = .001, Figure 2). The study cohort was 59% male, with a median age of 55 years (interquartile range, 41-65 years). Baseline characteristics of included patients with respect to ECMO mode and presence of MES are presented in Table 1. Compared with patients with TCD, those who lacked a TCD examination had a greater rate of COVID-19 (75.6% vs 51.1%, *P* = .03, Table E1). Of 100 VA-ECMO patients, 42 (42.0%) had an intra-aortic balloon pump and 7 (7.0%) had a left ventricular assist device placed before or synchronously with ECMO cannulation.

**Table 1:** Demographics and medical history of patients on VA-ECMO and VV-ECMO undergoing TCD, with respect to ever having an MES recorded on at least 1 examination

Demographics and comorbidities	VA-ECMO			VV-ECMO		
	No MES (n = 65)	MES (n = 35)	*P* value	No MES (n = 43)	MES (n = 2)	*P* value
Age, y	56 (46-68)	60 (47-68)	.80	47 (39-57)	44 (30-57)	.79
Male	59 (59%)	33 (76.7%)	.06	31 (75.6%)	27 (60.0%)	.17
Race			.45			.86
White	60 (60.0%)	22 (51.2%)		18 (40.0%)	14 (34.2%)	
Black	29 (29.0%)	16 (37.2%)		13 (28.9%)	15 (36.6%)	
Hispanic	3 (3.0%)	0		11 (24.4%)	9 (22.0%)	
Asian	4 (4.0%)	1 (2.3%)		1 (2.2%)	2 (4.9%)	
Other	4 (4.0%)	4 (9.3%)		2 (4.4%)	1 (2.4%)	
Hypertension	48 (73.9%)	27 (77.1%)	.81	18 (41.9%)	1 (50.0%)	1.00
Hyperlipidemia	37 (56.9%)	19 (54.3%)	.84	15 (34.9%)	1 (50.0%)	1.00
Atrial fibrillation	19 (29.3%)	12 (34.3%)	.65	0	0	–
Congestive heart failure	18 (27.7%)	13 (37.1%)	.37	0	0	–
Diabetes	22 (33.9%)	8 (22.9%)	.36	6 (14.0%)	0	1.00
Chronic kidney disease (stage 3+)	10 (15.4%)	4 (11.4%)	.77	2 (4.7%)	0	1.00
Intracerebral hemorrhage	0	1 (2.9%)	.35	0	0	–
Ischemic stroke	6 (9.2%)	2 (5.7%)	.71	2 (4.7%)	0	1.00
Anticoagulation before index admission	14 (21.5%)	12 (34.3%)	.23	3 (7.0%)	0	1.00
Antiplatelet therapy before index admission	28 (43.1%)	16 (45.7%)	.84	5 (11.6%)	0	1.00
Precannulation Glasgow Coma Scale score	15 (14-15)	15 (14-15)	.96	11 (3-15)	13 (11-15)	.61
VA-ECMO indication						
Postcardiotomy shock	29 (44.6%)	18 (51.4%)	.52			
Intraoperative cardiotomy shock	14 (21.5%)	8 (22.9%)	1.00			
Postoperative cardiotomy shock	15 (23.1%)	10 (28.6%)	.63			
Cardiogenic shock	21 (32.3%)	13 (37.1%)	.66			
Acute ischemic cardiomyopathy	11 (16.9%)	6 (17.1%)	1.00			
Chronic ischemic cardiomyopathy	1 (1.5%)	1 (2.9%)	1.00			
Nonischemic cardiomyopathy	9 (13.9%)	6 (17.1%)	.77			
Postheart transplant	2 (3.1%)	3 (8.6%)	.34			
Bridge to heart transplant	0	1 (2.9%)	.35			
Cardiac arrhythmia	8 (12.3%)	0	.05			
Pulmonary embolism	4 (6.2%)	0	.30			
Distributive shock	1 (1.0%)	0	1.00			
VV-ECMO indication						
Acute respiratory distress syndrome				39 (90.7%)	1 (50.0%)	.21
Bacterial pneumonia				5 (11.6%)	0	1.00
COVID-19 pneumonia				23 (53.5%)	0	.23
Other viral pneumonia				2 (4.7%)	0	1.00
Fungal pneumonia				2 (4.7%)	0	1.00
Pulmonary embolism				2 (4.7%)	0	1.00
Septic shock				2 (4.7%)	0	1.00
Aspiration pneumonitis				2 (4.7%)	0	1.00
Pancreatitis				0	1 (50.0%)	.05
Other				1 (2.4%)	0	1.00
Postlung transplant				2 (4.7%)	1 (50.0%)	.13
Bridge to lung transplant				2 (4.7%)	0	1.00

Results are presented as n (%) or median (interquartile range) as appropriate. *VA*, Venoarterial; *ECMO*, extracorporeal membrane oxygenation; *VV*, venovenous; *MES*, microembolic signal; *COVID-19*, coronavirus disease 2019; *TCD*, transcranial Doppler.

### TCD Microembolic Signals (MES)

**Figure 3:**
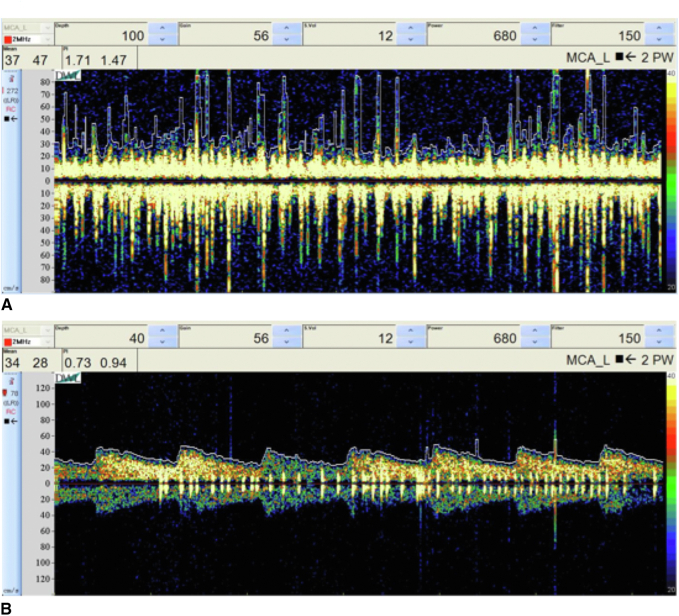
Examples of transcranial Doppler (*TCD*) waveforms exhibiting high microembolic signals in the left middle cerebral artery in patients receiving (A) venoarterial (*VA*) and (B) venovenous (*VV*) extracorporeal membrane oxygenation (*ECMO*). *MCA*, Middle cerebral artery.

MES were observed in 35 (35.0%) patients on VA-ECMO and 2 (4.4%) patients on VV-ECMO (*P* < .001), in 46 (24.6%) and 2 (3.1%) TCD examinations, respectively. Representative waveforms of TCD examinations in patients with high numbers of MES in VA- and VV-ECMO are presented in Figure 3.

### VA-ECMO

In patients on VA-ECMO with MES, mild grading was most common (65.2%), 7 examinations (15.2%) exhibited a moderate number of MES, and 9 examinations (19.6%) were severe. When MES were present in VA-ECMO, a median of 4 (2-16) hits were detected in each examination, affecting the following vessels: right MCA M1 segment (23.9%), right ACA A1 segment (13.0%), right ICA C1 segment (10.9%), right VrA (15.2%), left MCA M1 segment (28.3%), left ACA A1 segment (17.4%), left ICA C1 segment (32.6%), left VrA (17.4%), and BA proximal segment (28.3%). MES were present in 29.4% of patients on VA-ECMO without additional cardiac support, compared with 38.1% with intra-aortic balloon pump and 57.1% with left ventricular assist device, but these differences were not statistically significant (*P* = .39 and *P* = .20, respectively). Presence or number of MES was not associated with VA-ECMO cannulation mode (23.4% MES positivity in peripheral cannulation vs 25.8% in central, *P* = .74).

Although common (63.6%), the presence, position, or distance of clot or fibrin in the ECMO circuit at the time of TCD was not associated with a more frequent presence or greater grade of MES, and nor was a high gradient across the oxygenator (greater than 20 mm Hg). No significant correlation was found between presence or grade of MES and any studied hemodynamic (systolic blood pressure, diastolic blood pressure, mean arterial pressure), laboratory (arterial blood gas results, hemoglobin/hematocrit, platelet, fibrinogen, aPTT), or ECMO (flow and speed) parameters at the time of TCD (Table E2).

ABI occurred in 38% of patients on VA-ECMO and 31.1% of patients on VV-ECMO, whereas arterial thromboembolic events occurred in 37% of patients on VA-ECMO and 26.7% of patients on VV-ECMO. Neither ABI nor a composite event of arterial thromboembolic complications was associated with a more frequent presence or greater grade of MES on TCD (Table 2). Multivariable logistic regression of this composite arterial thromboembolic outcome, adjusting for variables with *P* values less than .20 on univariate analysis (sex and cardiac arrhythmia as indication for ECMO), similarly demonstrated no significant association. Median modified Rankin scale score at discharge was greater in patients on VA-ECMO with MES positivity (6 [5-6] vs 5 [5-6]), but this difference was not statistically significant (*P* = .37).

**Table 2:** Course and outcomes of patients on VA-ECMO and VV-ECMO undergoing TCD, with respect to ever having a MES recorded on at least 1 examination

ECMO course	VA-ECMO			VV-ECMO		
	No MES (n = 65)	MES (n = 35)	*P* value	No MES (n = 43)	MES (n = 2)	*P* value
Cannulation			.21			–
Central	26 (40.0%)	19 (54.3%)		–	–	
Peripheral	39 (60.0%)	16 (45.7%)		43 (100%)	2 (100%)	
Duration, d, median (IQR)	5 (3-10)	7 (3-11)	.40	25 (14-44)	10 (6-13)	.40
Transfusion products, units, median (IQR)						
Packed red blood cells	13 (5-20)	13 (6-21)	.72	9 (6-20)	12 (0-24)	.67
Platelets	3 (1-7)	2 (1-9)	.92	1 (0-3)	15 (0-30)	.55
Fresh-frozen plasma	2 (0-7)	2 (0-7)	1.00	0 (0-1)	6 (0-11)	.27
Cryoprecipitate	1 (0-2)	0 (0-2)	.97	0 (0-2)	5 (0-9)	.59
Inotrope or vasopressor requirement	63 (96.9%)	34 (97.1%)	1.00	37 (86.1%)	1 (50.0%)	.29
Hypertension requiring vasodilator	4 (6.2%)	4 (11.4%)	.45	3 (7.0%)	0	1.00
Arrhythmia	17 (26.2%)	13 (37.1%)	.26	6 (16.3%)	1 (50.0%)	.33
Hemolysis	28 (43.1%)	11 (31.4%)	.29	12 (27.9%)	1 (50.0%)	.50
Bloodstream infection	15 (23.1%)	6 (17.1%)	.61	19 (44.2%)	0	.50
Continuous renal-replacement therapy	41 (63.1%)	22 (62.9%)	1.00	20 (46.5%)	1 (50.0%)	1.00
Circuit clot	37 (56.9%)	21 (60.0%)	.83	33 (76.7%)	2 (100.0%)	1.00
Circuit fibrin	36 (55.4%)	22 (62.9%)	.53	36 (83.7%)	2 (100.0%)	1.00
Outcomes				0		
Neurologic				0		
Ischemic stroke	16 (24.6%)	4 (11.4%)	.19	4 (9.3%)	0	1.00
Intracerebral hemorrhage	6 (9.2%)	1 (2.9%)	.42	7 (16.3%)	0	1.00
Subarachnoid hemorrhage	5 (7.7%)	0	.16	7 (16.3%)	0	1.00
Subdural hemorrhage	4 (6.2%)	1 (2.9%)	.66	1 (2.3%)	0	1.00
Hypoxic ischemic brain injury	6 (9.2%)	4 (11.4%)	.74	1 (2.3%)	0	1.00
Cerebral edema	4 (6.2%)	2 (5.7%)	1.00	2 (4.7%)	0	1.00
Seizure	3 (4.6%)	4 (11.4%)	.24	2 (4.7%)	0	1.00
Brain death	1 (1.5%)	1 (2.9%)	1.00	3 (6.9%)	0	1.00
Coma (Glasgow Coma Scale <8)	18 (27.7%)	8 (22.9%)	.81	2 (4.7%)	1 (50.0%)	.13
Stroke/ICH/HIBI/BD	26 (40.0%)	9 (25.7%)	.19	10 (23.3%)	0	1.00
Stroke/ICH/HIBI/BD/SDH/SAH	28 (43.1%)	10 (28.6%)	.20	14 (32.6%)	0	1.00
Modified Rankin Scale score at discharge	5 (5-6)	6 (5-6)	.37	6 (4-6)	4 (1-6)	.73
Thromboembolic						
Intracardiac thrombus	2 (3.1%)	1 (2.9%)	1.00	0	1 (50.0%)	.05
Pulmonary embolism	4 (6.2%)	3 (8.6%)	.69	5 (11.6%)	0	1.00
Deep venous thrombosis	9 (13.9%)	1 (2.9%)	.16	8 (18.6%)	0	1.00
Heparin-induced thrombocytopenia	2 (3.1%)	2 (5.7%)	.61	6 (14.0%)	0	1.00
Arterial cannula clot	3 (4.6%)	0	.55	3 (7.0%)	0	1.00
Any thromboembolic event	38 (58.5%)	17 (48.6%)	.40	18 (41.9%)	1 (50.0%)	1.00
Arterial ischemic/thromboembolic (stroke, HIBI, intracardiac, PE, arterial clot)	27 (41.5%)	10 (28.6%)	.28	11 (25.6%)	1 (50.0%)	.47
Hemorrhagic				0		
Surgical site	21 (32.3%)	13 (37.1%)	.66	6 (14.0%)	0	1.00
Gastrointestinal	8 (12.3%)	5 (14.3%)	.77	10 (23.3%)	0	1.00
Cannula site	12 (18.5%)	7 (20.0%)	1.00	6 (14.0%)	0	1.00
Disseminated intravascular coagulation	4 (6.2%)	1 (2.9%)	.66	2 (4.7%)	0	1.00
Pulmonary	5 (7.7%)	3 (8.6%)	1.00	5 (11.6%)	1 (50.0%)	.25
Any bleeding event	40 (61.5%)	23 (65.7%)	.83	32 (74.4%)	1 (50.0%)	.47
Death	43 (66.2%)	28 (80.0%)	.17	22 (51.2%)	1 (50.0%)	1.00
Withdrawal of life-sustaining therapy	36 (55.4%)	26 (74.3%)	.08	20 (46.5%)	1 (50.0%)	1.00
Reason for withdrawal of life-sustaining therapy						
Family request	32 (49.2%)	24 (68.6%)	.09	12 (27.9%)	1 (50.0%)	.50
Hemorrhage	7 (10.8%)	3 (8.6%)	1.00	1 (2.3%)	0	1.00
Multiorgan failure	18 (27.7%)	14 (40.0%)	.26	6 (14.0%)	0	1.00
Ischemic stroke	2 (3.1%)	2 (5.7%)	.61	0	0	–
Hemorrhagic stroke	0	1 (2.9%)	.35	1 (2.3%)	0	1.00
Discharge location			.18			.46
Death	43 (66.2%)	28 (80.0%)		24 (55.8%)	1 (50.0%)	
Hospice	0	0		0	0	
Long-term acute care	0	0		0	0	
Skilled nursing facility	6 (9.2%)	1 (2.9%)		1 (2.3%)	0	
Acute rehabilitation	9 (13.9%)	1 (2.9%)		14 (32.6%)	0	
Home	7 (10.8%)	5 (14.3%)		4 (9.3%)	1 (50.0%)	

Results are presented as n (%) or median (interquartile range) as appropriate. *VA*, Venoarterial; *ECMO*, extracorporeal membrane oxygenation; *VV*, venovenous; *MES*, microembolic signal; *IQR*, interquartile range; *ICH*, intracranial hemorrhage; *HIBI*; hypoxic–ischemic brain injury; *BD*, brain death; *SDH*, subdural hematoma; *SAH*, subarachnoid hemorrhage; *PE*, pulmonary embolism; *TCD*, transcranial Doppler.

### VV-ECMO

Only 2 (4.7%) patients on VV-ECMO had a TCD with MES, and both were of severe grading with showers of emboli, affecting only the BA proximal segment in one case and bilateral ACA A1 and ICA C1 segments in the other. Of these 2 patients, neither had ABI but one had an intracardiac thrombus and ultimately was removed from ECMO.

Acute or subacute ischemic stroke was the most common neurologic event, identified in 24 patients (20 without MES and 4 with MES). The most common presumed etiology of ischemic stroke according to TOAST (trial of ORG 10172 in acute stroke treatment) classification^16^ was cardioembolism in all but one case of presumed small vessel occlusion. Most strokes appeared as multifocal areas of infarction within the supratentorial and/or infratentorial brain.

## Discussion

In this large cohort of patients with ECMO with TCD examinations, the presence of MES was detected in more than one-third of patients on VA-ECMO and approximately 5% of patients on VV-ECMO. There are few studies characterizing the prevalence of MES ECMO, and fewer still that explore their possible clinical significance. The long-standing implementation of a neuromonitoring protocol including TCD at our institution permits the examination of TCD in relation to numerous clinical covariates and neurologic outcomes. Here, ABI was found in 38.0% and 31.1% of patients on VA-ECMO and VV-ECMO, respectively, and arterial embolic events occurred in 37.0% and 26.7%, respectively. Despite several potential sources of emboli to the cerebral circulation, this study did not identify significant associations with MES, nor were MES associated with greater frequency of ABI or ischemic stroke, although nearly all ischemic strokes were presumed to be embolic in etiology.

TCD is the only technique available for the assessment of MES, which have been described in a number of cardiovascular conditions with widespread implications including neurologic, including carotid artery stenosis, atrial fibrillation, myocardial infarction, prosthetic heart valves, valvular stenosis, carotid surgery, open heart surgery, valve surgery, angiography, and angioplasty.^17^, ^18^, ^19^, ^20^, ^21^ Only 1 previous study investigated the prevalence and interpretation of TCD MES in ECMO,^12^ where thrombosis in the ECMO circuit, decannulation procedures, and intracardiac stasis in cardiogenic shock were all potential sources of embolization to the cerebral circulation.^22^^,^^23^

Marinoni and colleagues^12^ in 2016 first reported the prevalence of MES in 11 patients on VV-ECMO and 42 VA-ECMO: 26.2% and 81.8%, respectively, a rate considerably greater than here reported. Similar to this study, most recorded clinical parameters, such as ECMO flow or speed and aPTT, were not associated with the presence of MES. Left ventricular ejection fraction was inversely correlated with MES grading in patients on VA-ECMO (*P* = .037). A similar inverse relationship was described in this study (35% [10%-60%] for TCDs without MES vs 20% [10%-55%] with MES), but this difference was not significant. However, echocardiography is not part of the routine monitoring protocol for ECMO patients at our institution, and, as such, is not consistently available for many TCD time points. Although this study did not identify significant associations with MES, congestive heart failure, arrhythmias, and central cannulation were all more common in patients on VA-ECMO with MES compared with without, suggesting potential for cardioembolic events.

Marinoni and colleagues^12^ reported no association between MES presence or grading with clinical outcomes, up to 6 months after discharge, although the sample size was small. Similarly, in this larger study the presence or number of MES on TCD in both patients receiving VA-ECMO and VV-ECMO was not significantly associated with either ABI or thromboembolic events, considered both as composite and individual events. The incidence of acute ischemic stroke was more common in patients on VA-ECMO without MES than with MES (24.6% vs 11.4%), which was not expected, given the postulated association of microemboli with ischemic stroke, particularly with VA-ECMO and high rate of thrombotic complications in ECMO patients.^2^^,^^3^^,^^24^, ^25^, ^26^ Possible explanations for lack of clinical significance of TCD MES in relation to ischemic stroke or ABI include small sample size, relatively short duration of TCD monitoring, and lack of neuroimaging (performed in 81.8%), and that decannulation procedures were not monitored with TCD. We may speculate, therefore, that many embolic events were not captured with brief TCD monitoring and that MES remain an important surrogate measure of risk of arterial thrombotic events in this population.

Standardized neuromonitoring of patients receiving ECMO is a relatively novel practice that is changing the neurologic care of these high-risk patients. Recently, we described a multimodal neuromonitoring protocol composed of serial neurologic examinations, neuroimaging (computed tomography/magnetic resonance imaging), electroencephalography, and transcranial ultrasound, which demonstrated greater capacity to detect ABI and facilitate prompt, targeted interventions such as anticoagulation, supplemental oxygen, and blood pressure control.^11^ This technique has led to greater detection and improved management of ABI on ECMO.^27^ At this point, however, the clinical decision-making impacted by discovery of MES is unclear. Further studies will be needed to better describe the relationship of oxygenator clot to MES, and the impact of decannulation on MES.

### Limitations

This study was conducted at a single center, which reduces generalizability despite a sizable cohort. Although the high interobserver variability of TCD performance was minimized by a study design with 2 experienced technologists,^28^ use of longer duration of TCD monitoring using headsets or robotic TCD may have increased detection rates of MES. TCD monitoring is also moving toward automatic recording, but this has yet not been validated in patients in ECMO, where significant facial edema, neck line access, and positioning are complicating factors. This study was also subject to incomplete capture of eligible patients for reasons described previously. Also, the evaluation of “macroscopic” neurologic outcomes such as stroke is limited with respect to the study outcomes (MES on ECMO and neurologic consequences thereof). There were several imitations in data collection, including a lack of imaging and longitudinal follow-up in surviving patients to detect less severe long-term neurologic deficits that could be related to MES. Residual cardiac function while on VA-ECMO could also impact the degree of anterograde and retrograde cerebral blood flow, thus influencing the presence or number of MES. Unfortunately, putative metrics to reflect residual cardiac function on VA-ECMO, such as left ventricular ejection fraction or arterial waveform pulsatility, were not available in a reliable and consistent manner and thus could not be assessed within this study. Although the vast majority of TCD examinations were performed early on ECMO, a few performed as clinically indicated later in the course during the ECMO weaning process could have similarly affected the degree of cerebral blood flow and subsequent MES evaluation. Perfusion gradient and pump clot or fibrin assessments were also challenging, and have been modified over the course of the study to better standardize and completely capture arterial or venous cannula and oxygenator clots, which are often difficult to detect and vary considerably over time.

### Conflict of Interest Statement

A.G. and S.M.C. are supported by the National Institutes of Health (NIH). W.C.Z. is supported by the NIH and has received consulting fees from C.R. Bard, Inc, outside of the area of work commented on here. All other authors reported no conflicts of interest.

The *Journal* policy requires editors and reviewers to disclose conflicts of interest and to decline handling or reviewing manuscripts for which they may have a conflict of interest. The editors and reviewers of this article have no conflicts of interest.

## Figures and Tables

**Table E1:** Baseline characteristics of patients receiving VA-ECMO and VV ECMO, included or excluded based on availability of TCD study

Demographics and comorbidities	VA-ECMO			VV-ECMO		
	TCD (n = 100)	No TCD (n = 43)	*P* value	TCD (n = 45)	No TCD (n = 41)	*P* value
Age, y	57 (47-68)	60 (44-65)	.99	47 (39-57)	50 (39-56)	.79
Male	59 (59%)	33 (76.7%)	.06	31 (75.6%)	27 (60.0%)	.17
Race			.45			.86
White	60 (60.0%)	22 (51.2%)		18 (40.0%)	14 (34.2%)	
Black	29 (29.0%)	16 (37.2%)		13 (28.9%)	15 (36.6%)	
Hispanic	3 (3.0%)	0		11 (24.4%)	9 (22.0%)	
Asian	4 (4.0%)	1 (2.3%)		1 (2.2%)	2 (4.9%)	
Other	4 (4.0%)	4 (9.3%)		2 (4.4%)	1 (2.4%)	
Hypertension	75 (75.0%)	29 (67.4%)	.41	19 (42.2%)	16 (39.0%)	.83
Hyperlipidemia	56 (56.0%)	23 (53.5%)	.85	16 (35.6%)	13 (31.7%)	.82
Atrial fibrillation	31 (31.0%)	9 (20.9%)	.31	0	0	–
Congestive heart failure	31 (31.0%)	16 (37.2%)	.56	0	0	–
Diabetes	30 (30.0%)	16 (37.2%)	.44	6 (13.3%)	8 (19.5%)	.56
Chronic kidney disease (stage 3+)	14 (14.0%)	10 (23.3%)	.22	2 (4.4%)	1 (2.4%)	1.00
Intracerebral hemorrhage	1 (1.0%)	0	1.00	1 (2.2%)	0	1.00
Ischemic stroke	8 (8.0%)	4 (9.3%)	.75	2 (4.4%)	1 (2.4%)	1.00
Anticoagulation before index admission	26 (26.0%)	7 (16.3%)	.28	3 (6.7%)	4 (9.8%)	.70
Antiplatelet therapy before index admission	44 (44.0%)	22 (51.2%)	.47	5 (11.1%)	4 (9.8%)	1.00
Pre-ECMO Glasgow Coma Scale score	15 (14-15)	15 (13-15)	.96	11 (3-15)	4 (3-15)	.06
ECMO support, d	6 (3-10)	5 (3-8)	.08	25 (13-41)	17 (10-34)	.42
Central cannulation	55 (55.0%)	31 (72.1%)	.06	0	0	–
VA-ECMO indication						
Postcardiotomy shock	47 (47.0%)	15 (34.9%)	.20			
Intraoperative cardiotomy shock	22 (22.0%)	5 (11.6%)	.17			
Postoperative cardiotomy shock	25 (25.0%)	10 (23.3%)	1.00			
Cardiogenic shock	34 (34.0%)	20 (26.5%)	.19			
Acute ischemic cardiomyopathy	17 (17.0%)	6 (14.0%)	.81			
Chronic ischemic cardiomyopathy	2 (2.0%)	4 (9.3%)	.07			
Nonischemic cardiomyopathy	15 (15.0%)	10 (23.3%)	.24			
Postheart transplant	5 (5.0%)	4 (9.3%)	.45			
Bridge to heart transplant	1 (1.0%)	2 (4.7%)	.22			
Cardiac arrhythmia	8 (8.0%)	2 (4.7%)	.72			
Pulmonary embolism	4 (4.0%)	0	.32			
Distributive shock	1 (1.0%)	0	1.00			
VV-ECMO indication						
Acute respiratory distress syndrome				40 (88.9%)	37 (90.2%)	1.00
Bacterial pneumonia				5 (11.1%)	4 (9.8%)	1.00
COVID-19 pneumonia				23 (51.1%)	31 (75.6%)	**.03**
Other viral pneumonia				2 (4.4%)	0	.50
Fungal pneumonia				2 (4.4%)	0	.50
Pulmonary embolism				2 (4.4%)	0	.50
Septic shock				2 (4.4%)	0	.50
Aspiration pneumonitis				2 (4.4%)	0	.50
Pancreatitis				1 (2.2%)	0	1.00
Other				1 (2.2%)	3 (7.3%)	.34
Postlung transplant				3 (6.7%)	3 (7.3%)	1.00
Bridge to lung transplant				2 (4.4%)	0	.50

Results presented as n (%) or median (interquartile range) as appropriate. *P* value in bold indicates statistically significant. *VA*, Venoarterial; *ECMO*, extracorporeal membrane oxygenation; *VV*, venovenous; *TCD*, transcranial Doppler; *COVID-19*, coronavirus disease 2019.

**Table E2:** Hemodynamic and laboratory parameters at the time of transcranial Doppler study while on VA-ECMO, with respect to the presence of MES

	No MES (n = 141)	MES (n = 46)	*P* value
Systolic blood pressure, mm Hg	98 (86-114)	93 (76-110)	.22
Diastolic blood pressure, mm Hg	62 (52-68)	60 (52-68)	.52
Mean arterial pressure, mm Hg	72 (66-82)	71 (62-77)	.06
Hemoglobin, g/dL	8.2 (7.7-9.0)	8.2 (7.6-9.4)	.72
Hematocrit, %	24.5 (22.8-26.6)	25.4 (23.1-27.8)	.23
Platelet count, k/μL	62 (43-94)	79 (59-97)	.30
Fibrinogen, mg/dL	292 (212-463)	300 (211-481)	.77
Activated partial thromboplastin time, s	48.3 (33.8-69.5)	51.7 (42.5-70.7)	.35
Arterial blood gas Pco_2_, mm Hg	38 (35-42)	39 (36-45)	.75
Arterial blood gas Po_2_, mm Hg	133 (94-202)	128 (109-221)	.81
Ejection fraction, %	35 (10-60)	20 (10-55)	.30
ECMO flow, L/min	4.3 (3.5-5.0)	4.5 (4.1-5.2)	.34
ECMO speed, rpm	3450 (3215-3760)	3600 (3450-3875)	.06

Results presented as N (%) or median (interquartile range) as appropriate. *MES*, Microembolic signals; *P**co*_*2*_, carbon dioxide tension; *P**o*_*2*_, oxygen tension; *ECMO*, extracorporeal membrane oxygenation; *VA-ECMO*, venoarterial extracorporeal membrane oxygenation.
